# The surgical workforce shortage and successes in retaining surgical trainees in Ethiopia: a professional survey

**DOI:** 10.1186/s12960-016-0126-7

**Published:** 2016-06-30

**Authors:** Miliard Derbew, Adam D. Laytin, Rochelle A. Dicker

**Affiliations:** Department of Surgery, School of Medicine, College of Health Sciences, Addis Ababa University, PO Box 5729, Addis Ababa, Ethiopia; Center for Global Surgical Studies, Department of Surgery, University of California San Francisco, San Francisco, CA USA; Department of Emergency Medicine, Oregon Health & Science University, Portland, OR USA

**Keywords:** Africa, Developing countries, Ethiopia, Surgical training, Surgical workforce

## Abstract

**Background:**

Medical workforce shortages represent a major challenge in low- and middle-income countries, including those in Africa. Despite this, there is a dearth of information regarding the location and practice of African surgeons following completion of their training. In response to the call by the WHO Global Code of Practice on the International Recruitment of Health Personnel for a sound evidence base regarding patterns of practice and migration of the health workforce, this study describes the current place of residence, practice and setting of Ethiopian surgical residency graduates since commencement of their surgical training in Ethiopia or in Cuba.

**Methods:**

This study presents data from a survey of all Ethiopian surgical residency training graduates since the programme’s inception in 1985.

**Results:**

A total of 348 Ethiopians had undergone surgical training in Ethiopia or Cuba since 1985; data for 327 (94.0 %) of these surgeons were collected and included in the study. The findings indicated that 75.8 % of graduates continued to practice in Ethiopia, with 80.9 % of these practicing in the public sector. Additionally, recent graduates were more likely to remain in Ethiopia and work within the public sector. The average total number of surgeons per million inhabitants in Ethiopia was approximately three and 48.0 % of Ethiopian surgeons practiced in Addis Ababa.

**Conclusions:**

Ethiopian surgeons are increasingly likely to remain in Ethiopia and to practice in the public sector. Nevertheless, Ethiopia continues to suffer from a drastic surgical workforce shortage that must be addressed through increased training capacity and strategies to combat emigration and attrition.

## Background

Diseases requiring surgical intervention have been estimated to account for 11 % of the global burden of disease [1]. Surgical care, which has been shown to be cost effective, could avert 20–40 % of this burden in low- and middle-income countries (LMICs), saving nearly 2 million lives annually [2–4]. Despite the magnitude of these estimates, surgery remains a neglected aspect of health systems development in LMICs [5, 6]. Modest estimates show that billions of people worldwide lack access to even basic surgical care [7] and only 3.5 % of all surgical operations worldwide are performed on the poorest 35 % of the world’s population [8].

An inadequate workforce remains a major challenge in surgical capacity building, with the emigration of health workers from LMICs often cited as a major impediment to workforce development [9–11]. Building surgical capacity is fundamental to addressing this burden of disease and is an important aspect of overall health systems strengthening in LMICs; however, the scope of the surgical workforce shortage is not well-quantified in many LMICs and effective strategies to increase the surgical workforce and promote local retention of surgeons have not been well tested [12]. The WHO Global Code of Practice on the International Recruitment of Health Personnel sets out guiding principles for addressing the shortage of health personnel and emphasizes the importance of gathering and sharing national data about international recruitment in achieving this goal [13].

Ethiopia is the second most populous country in sub-Saharan Africa. In 2009, 2152 physicians were practicing in Ethiopia, equating to one physician for every 42,000 inhabitants, compared to one physician for every 410 inhabitants in the United States [14]. According to the Human Resources for Health survey [15], in 2009, there were 140 practicing surgeons in Ethiopia, which is far from the projected requirement of 522 surgeons; additionally, it was estimated that 820 surgeons would be required by 2015. Despite these issues, there is a dearth of published data regarding Ethiopian physician attrition or emigration. The only available report on the physician workforce shortage in Ethiopia was published by Berhan in 2008, indicating that 73 % of Ethiopian physicians left the public sector to work in private hospitals or non-governmental organisations, or to live abroad [16]. Furthermore, most physicians practiced in urban settings, leading to a physician staff shortage in over 80 % of public hospitals outside Addis Ababa. Considering that over 80 % of Ethiopia’s population lives in rural areas, the uneven distribution of physicians is a major barrier to accessing healthcare services. Berhan’s study, however, did not specifically address workforce issues related to surgeons. Chao et al. [17] addressed the surgical infrastructure in Ethiopia and estimated an average of 4.2 surgeons per hospital based on a convenience sample of 20 hospitals; nevertheless, their study did not report a national count of surgeons or an estimate of the surgical workforce shortage.

Over the past 10 years, Ethiopia has undergone a rapid expansion in surgical training capacity, going from only one surgical residency program in 2004 to eight surgical residency programs in 2014. However, little has been reported with regards to the location and practice of Ethiopian surgeons following completion of their training. The present study aims to describe the current location, practice and setting of all Ethiopian general surgeons who graduated over the past 33 years in order to gain a better understanding of the attrition, retention and emigration of Ethiopian surgeons. The findings herein provide insight on the impact of expanding surgical residency programs on Ethiopia’s healthcare workforce shortage.

## Methods

### Study setting

Ethiopia has a population of 92 million people, more than 80 % of whom live in rural areas. Ethiopia’s per capita gross national income is US$ 453, with over 30 % of the population living below the international poverty line of US$ 1.25/day. Further, only 2.6 % of the gross domestic product is allocated to public spending on health [18, 19].

Until the 1980s, the only surgeons practicing in Ethiopia had been trained abroad. The Addis Ababa University Faculty of Medicine established the country’s first surgery residency at Tikur Anbessa Hospital in 1980, which remained the only surgery residency in the country until 2004. The Addis Ababa University surgical residency currently accepts 25 new residents per year, and these residents rotate through six public hospitals around the city. Since 2005, seven more surgical residency programs have been established, and subspecialty training in neurosurgery, urology, cardiothoracic surgery, plastic and reconstructive surgery, and paediatric surgery has commenced at Tikur Anbessa Hospital.

### Study design

A list of graduates of all eight surgical residency programs in Ethiopia between 1985 and 2013 was compiled from the administrative records of each institution’s residency program (Table 1). A small number of Ethiopian citizens pursued graduate medical education in Cuba during this time period; therefore, a list of Ethiopian citizens who graduated from surgical residencies in Cuba (including general surgery, neurosurgery, urology, and otorhinolaryngology) between 1985 and 2013 was also compiled. We were unable to identify any Ethiopian citizens who obtained residency training in surgery in any other countries from 1985 to 2013 who are currently practicing in Ethiopia.

**Table 1:** General surgery residency programs in Ethiopia

Name	Region	Year established	Current number of trainees ^a^	Model
Addis Ababa University	Addis Ababa	1980	100	University
Gondar University	Amhara	2004	23	University
Jimma University	Oromia	2008	28	University
Meleke University	Tigray	2012	23	University
St. Paul’s Millennium College	Addis Ababa	2013	15	University
Hawasa University	SNNPR	2014	4	University
Bahir Dar University	Amhara	2013	15	University
Soddo Christian Hospital	SNNPR	2005	8	Hospital
Total			216	

^a^ Anticipated graduation 2014–2017

*SNNPR* The Southern Nations, Nationalities and People’s Region

Note: Since 2004, seven new surgical residencies have been established in Ethiopia. However, Addis Ababa University remains the largest program in the country, matriculating 25 residents per year

Graduates were contacted directly by the study team via telephone and/or email to collect data about their current place of residence and employment. When attempts to directly contact graduates were unsuccessful, their colleagues were contacted as a secondary data source to gather information regarding these surgeons’ current employment, and these results were verified with other colleagues whenever possible. Additional attempts were made to verify these findings with Ministry of Health records; however, the records reviewed lacked updated information.

### Data analysis

Data were tabulated in a spreadsheet using Microsoft Excel and imported into STATA 13 for descriptive analysis. Bivariate analyses were performed using Fisher’s exact test and Pearson’s *x*^2^ test to evaluate the potential impacts of country of training and year of graduation on current place of residence and practice setting.

## Results

**Fig. 1:**
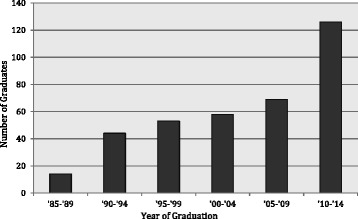
Number of Ethiopian graduates from Ethiopian and Cuban general surgery residency programs, 1985–2014

Between 1985 and 2013, 324 general surgeons graduated from residency training in Ethiopia; data were collected on 306 (94.4 %) since the study team was unable to contact the remaining surgeons or to find colleagues who knew of their current place of residence or practice setting. Of the graduates of Ethiopian residencies, the majority (90.5 %; *n* = 277) graduated from Addis Ababa University, followed by Jimma University (4.0 %; *n* = 12), Gondar University (3.6 %; *n* = 11), and the Pan-African Academy of Christian Surgeons (1.9 %; *n* = 6). The number of graduates per year steadily increased over the past 30 years (Fig. 1).

An additional 24 Ethiopian surgeons graduated from residency training in Cuba, including 15 general surgeons, four urologists, three head and neck surgeons, and two neurosurgeons. Data were collected on all of the Ethiopian surgeons who trained in Cuba, leading to a total of 330 survey participants. One Ethiopian and one Cuban graduate were found to be retired, and one Cuban graduate deceased, resulting in a sample size of 327 surgeons.

**Fig. 2:**
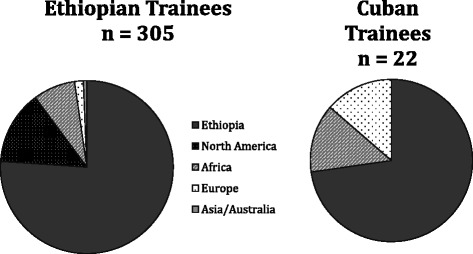
Current place of residence of Ethiopian surgical residency graduates trained in Ethiopia and Cuba

**Fig. 3:**
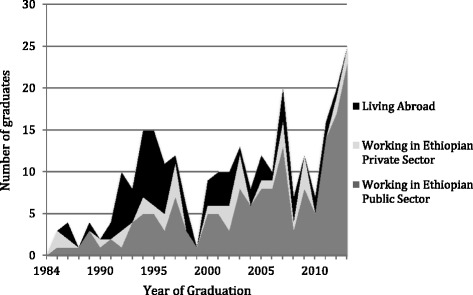
Ethiopian surgical residency graduates by year, place of residence and practice sector

Of the 327 graduates for whom data were available, 248 were resident in Ethiopia (75.8 %), with the 42 living in North America (12.9 %), 27 elsewhere in Africa (8.3 %), seven in Europe (2.1 %), and two in Australia and Asia (0.6 %). Surgeons who trained in Ethiopia or in Cuba were equally likely to be living in Ethiopia at the time of the study (76 % vs. 73 %, *P* = 0.72; Fig. 2). More recent graduates were more likely to be practicing in Ethiopia and within the Ethiopian public sector (Fig. 3).

Of the 248 graduates living in Ethiopia, employment data were available for 227 (91.5 %). Of these, 225 were currently practicing surgery (99.1 %). The region and type of practice of graduates are shown in Table 2. The majority of surgeons were employed in the capital city, Addis Ababa (*n* = 108; 48.0 %), despite only 3.7 % of the country’s population being concentrated in the city. Overall, 80.9 % (*n* = 182) of surgeons practicing in Ethiopia practiced primarily in the public sector, although many of these surgeons engaged in “dual practice” by also working in the private sector to augment their income. The private and missionary/non-governmental organization sectors remained non-existent in many regions of the country. Finally, 52.9 % (*n* = 119) of graduates worked, at least part time, at academic hospitals and were involved in surgical resident training.

**Table 2:** Current region and practice setting of Ethiopian surgeons practicing in Ethiopia

Region	Public, *n* (%)	Private, *n* (%)	Missionary/NGO, *n* (%)	Total, *n*
Addis Ababa	83 (77)	22 (20)	3 (3)	108
Afar	2 (100)	0 (0)	0 (0)	2
Amhara	27 (87)	4 (13)	0 (0)	31
Dire Dawa	2 (100)	0 (0)	0 (0)	2
Harari	3 (100)	0 (0)	0 (0)	3
Oromia	28 (80)	3 (9)	4 (11)	35
SNNPR	19 (83)	2 (9)	2 (9)	23
Somali	1 (100)	0 (0)	0 (0)	1
Tigray	16 (94)	1 (6)	0 (0)	17
Unknown	1 (33)	1 (1)	1 (1)	3
Total	182 (81)	33 (15)	10 (4)	225

*NGO* Non-governmental organization; *SNNPR* The Southern Nations, Nationalities and People’s Region

## Discussion

The WHO Global Code of Practice on the International Recruitment of Health Personnel states that, “*Member States should strive, to the extent possible, to create a sustainable health workforce and work towards establishing effective health workforce planning, education and training, and retention strategies that will reduce their need to recruit migrant health personnel*” [13]. This is the first study of its kind to address these issues among surgeons in Ethiopia by striving to describe the current place of residence, practice and setting of Ethiopian graduates of surgical residencies.

The present study demonstrates that the majority of Ethiopian surgical trainees continue to live in Ethiopia and practice in the public sector. Furthermore, the proportional retention of surgeons in Ethiopia and of those serving in public sector hospitals has steadily increased since the inception of formal general surgery training programs in Ethiopia. The findings herein indicate that 75.8 % of Ethiopian general surgery trainees continue to practice in Ethiopia and that 80.9 % of these do so in the public sector. These figures are markedly higher than those indicated by Berhan, who found that from 1987 to 2006 only 27 % of Ethiopian physicians continued to practice in the public sector [16].

Several factors likely contribute to the low surgeon attrition rate in Ethiopia. One possible explanation is the impact exerted by the Ethiopian surgical faculty, who continue to work in the public sector themselves, thus serving as role models for trainees. Another possible explanation is the evolution of the private sector, which allows an increasing number of surgeons to engage in dual practice, maintaining their principal affiliation at a public hospital while augmenting their salary with a supplementary private practice. While dual practice reduces the time spent by surgeons in public hospitals, where they may have the greatest impact on the country’s burden of surgical disease, it combats emigration and makes practicing in Ethiopia financially sustainable.

In addition, over the past 10 years, new programs for subspecialty training, including paediatric surgery, cardiothoracic surgery, urology, neurosurgery, and orthopaedic surgery, have been developed, thus allowing more trainees to establish a unique niche and contributing to greater job satisfaction among surgeons. Finally, residency training in Ethiopia is partially funded through public sponsorship; therefore, many of the trainees who graduated within the past 10 years are obliged to remain in Ethiopia and practice in the public sector.

Ethiopia continues to have a marked shortage of general surgeons, with only 248 general surgeons, compared to the predicted need for 820. Further, this shortage is most pronounced in rural regions, which have an average of only one surgeon per million inhabitants or less (Table 3) [15]. The data presented in Table 3 demonstrate the tremendous disparity in distribution of surgeons throughout Ethiopia. Two rural regions (Benishangul-Gumuz and Gambella), which have a joint population of approximately 1.4 million people, have no residency-trained Ethiopian surgeons practicing there. While the number of surgeons is projected to increase dramatically with the continued expansion of newly established residency programs, there is ongoing uncertainty about the best way to meet Ethiopia’s surgical workforce demands. One such option, which has been successful in other contexts, is the establishment of rural practice pipelines, which recruit and support medical students interested in practicing in underserved regions [20, 21]. However, the lack of equipment and infrastructure necessary for surgical practice remains a major challenge at many hospitals around the country [17].

**Table 3:** Surgeons per region in Ethiopia

Region	Population [27]	Number of surgeons	Surgeons per 100,000 population
Addis Ababa	3,041,002	109	3.58
Affar	1,602,995	2	0.12
Amhara	18,866,002	36	0.19
Benishangul-Gumuz	982,004	0	0
Dire Dawa	387,000	5	1.29
Gambella	385,997	0	0
Harari	210,000	3	1.43
Oromia	31,294,992	39	0.12
Somali	5,148,989	1	0.02
SNNPR	17,359,008	27	0.16
Tigray	4,929,999	19	0.39
Total	84,320,987	248	0.29

*SNNPR* The Southern Nations, Nationalities and People’s Region

Although emigration and attrition among Ethiopian surgeons occurs less frequently than would be expected, this study shows that these phenomena remain real challenges in addressing Ethiopia’s surgical workforce shortages. Strategies have been described to encourage African doctors to remain in the settings where their services are most needed [22, 23]. In addition, some African countries have focused attention on training non-surgeons to perform basic, life-saving operations [24]. This approach has been successful in rural Ethiopia, although more complex operations continue to demand the expertise of fully trained surgeons and subspecialists [25].

Another approach to the surgical workforce shortage is a shift from urban, university-based training to rural, hospital-based training as suggested in Article 5.5 of the WHO Global Code of Practice, which calls on Member States to “*consider strengthening educational institutions to scale up the training of health personnel and developing innovative curricula to address current health needs*” [13]. This approach has been undertaken in Ethiopia by the Pan-African Academy of Christian Surgeons at Soddo Christian Hospital in southern Ethiopia, with great success in retaining graduates in rural and underserved areas [26]. There are many benefits to a hospital-based surgical residency. Foremost, trainees remain in their home communities, encouraging them to stay in rural areas following their training. These programs may also be easier and less expensive to scale up than university-based programs. Hosting a residency program requires training hospitals to be accredited through a process that ensures adequate infrastructure and human resources, thus encouraging rural hospitals to improve their capacity. However, the Pan-African Academy of Christian Surgeons’ model, which relies on significant financial and workforce support from foreign donors and volunteers, may not be scalable and therefore more government support for rural surgical training is urgently required.

University-based training programs remain an important component of surgical education in Ethiopia, training academic surgeons and subspecialists who will eventually lead surgical education, research and innovation. However, university-based training programs often require residents to migrate long distances from their homes to train in urban centres, and graduates are often reluctant to return to rural settings following completion of their training. Further, as training programs expand, trainees may not receive adequate hands-on experience or supervision during their residencies. As the volume of surgical training in Ethiopia continues to increase, it is crucial to ensure the continued quality of training through standardized curricula and board examinations, and to guarantee that Ethiopian surgeons have access to the physical and financial resources and infrastructure required to effectively care for their patients.

Although the development of the private medical sector in Ethiopia likely helps to combat surgeon emigration and attrition, it is unclear how much private sector surgery addresses the true burden of diseases requiring surgical care in LMICs. Important topics for further research include a comparative quality assessment of different resident training models, a more detailed characterization of the professional activities of surgeons practicing in Ethiopia, an investigation into the factors that influence the career decisions of Ethiopian surgeons, and an analysis of the clinical skills of surgeons trained in Ethiopia. In addition, article 5.7 of the WHO Global Code of Practice states that, “*Member States should consider adopting measures to address the geographical maldistribution of health workers and to support their retention in underserved areas*” [13]. Effective means of incentivizing, recruiting and retaining rural surgeons deserves further attention. These findings will help ensure that surgical training in Ethiopia truly addresses the country’s burden of disease.

There were several significant limitations to this study. Firstly, it relied on second-hand reporting of graduates’ current location and practice when these could not be successfully contacted directly. The weak medical infrastructure and lack of a robust alumni network for surgical trainees in Ethiopia made a more traditional data collection strategy unfeasible. Nevertheless, we were able to confirm our findings for many surgeons living in Ethiopia. On the other hand, the lack of reliable contact information limited our ability to learn about the current practice models of Ethiopian surgeons practicing abroad. In addition, data were not collected on Ethiopian citizens who trained in surgery in countries other than Ethiopia or Cuba or non-Ethiopian surgeons practicing in Ethiopia. However, we believe that the latter constitute a very small portion of the surgical workforce in Ethiopia. Finally, many public sector surgeons in Ethiopia are believed to have dual practice in the private sector to augment their income, yet we were unable to quantify the scope of this phenomenon herein.

## Conclusions

Over the past 33 years, 348 Ethiopians have completed post-graduate training in surgery. Of these graduates, the majority have remained in Ethiopia and continue to practice in the public sector. Despite these recent advances, Ethiopia continues to face a marked surgical workforce shortage. The WHO Global Code of Practice calls for a sound evidence base to inform the formulation of effective policies and plans on the health workforce [13]. In particular, addressing surgical workforce shortages in Ethiopia requires concerted efforts to expand surgical training capacity while maintaining high standards for quality of education in order to encourage graduates to remain in Ethiopia and to incentivize surgeons to practice in settings where they can provide maximal benefit for the population. Since the retention of surgeons in Ethiopia surpasses that of other physicians, labour market research addressing this success may offer invaluable lessons for other medical specialty communities in LMICs striving to reverse patterns of emigration.

## Abbreviation

LMICs: low- and middle-income countries
